# Personality, lifestyle and job satisfaction: causal association between neuroticism and job satisfaction using Mendelian randomisation in the UK biobank cohort

**DOI:** 10.1038/s41398-020-0691-3

**Published:** 2020-01-21

**Authors:** Gull Rukh, Junhua Dang, Gaia Olivo, Diana-Maria Ciuculete, Mathias Rask-Andersen, Helgi Birgir Schiöth

**Affiliations:** 1 Department of Neuroscience, Functional Pharmacology, Uppsala University, Uppsala, Sweden; 2 Department of Immunology, Genetics and Pathology, Medical Genetics and Genomics, Uppsala University, Uppsala, Sweden; 3 Institute for Translational Medicine and Biotechnology, Sechenov First Moscow State Medical University, Moscow, Russia

**Keywords:** Psychology, Genetics

## Abstract

Job-related stress has been associated with poor health outcomes but little is known about the causal nature of these findings. We employed Mendelian randomisation (MR) approach to investigate the causal effect of neuroticism, education, and physical activity on job satisfaction. Trait-specific genetic risk score (GRS) based on recent genome wide association studies were used as instrumental variables (IV) using the UK Biobank cohort (*N* = 315,536). Both single variable and multivariable MR analyses were used to determine the effect of each trait on job satisfaction. We observed a clear evidence of a causal association between neuroticism and job satisfaction. In single variable MR, one standard deviation (1 SD) higher genetically determined neuroticism score (4.07 units) was associated with −0.31 units lower job satisfaction (95% confidence interval (CI): −0.38 to −0.24; *P* *=* 9.5 × 10^−20^). The causal associations remained significant after performing sensitivity analyses by excluding invalid genetic variants from GRS_Neuroticism_ (*β*(95%CI): −0.28(−0.35 to −0.21); *P* *=* 3.4 x 10^−15^). Education (0.02; −0.08 to 0.12; 0.67) and physical activity (0.08; −0.34 to 0.50; 0.70) did not show any evidence for causal association with job satisfaction. When genetic instruments for neuroticism, education and physical activity were included together, the association of neuroticism score with job satisfaction was reduced by only −0.01 units, suggesting an independent inverse causal association between neuroticism score (*P* = 2.7 x 10^−17^) and job satisfaction. Our findings show an independent causal association between neuroticism score and job satisfaction. Physically active lifestyle may help to increase job satisfaction despite presence of high neuroticism scores. Our study highlights the importance of considering the confounding effect of negative personality traits for studies on job satisfaction.

## Introduction

On average, during the economically active period, individuals spend one third of their lifetime at work. Experiencing stress at the workplace is associated with increased all-cause mortality^1^ and negative health effects, particularly cardiovascular disease^2^. In contrast, job satisfaction has been consistently shown to be associated with job performance and organisational citizenship behaviours, as employees who are satisfied with their jobs are the driving force for healthy and productive companies^3^. Therefore, in recent decades, job satisfaction has been the most studied parameter in working life studies as a great emphasis has been placed on the value of health at the workplace^4^. Meanwhile, meta-analyses on studies related to job satisfaction and well-being also suggested that job satisfaction is an important predictor of physical and psychological health^5^, as well as subjective well-being^6^.

Prior research has identified many potential antecedents of job satisfaction, including both individual factors such as personality traits and organisational factors such as empowerment and workload^7^. Among the dispositional factors, the Big-Five personality traits are widely accepted and the most studied ones^8^. A recent meta-analysis showed that neuroticism was the strongest predictor of job satisfaction among the Big-Five (*r* = −0.29)^9^. Meanwhile, people’s educational level has also been found to be associated with job satisfaction, which was explained by assuming that those with higher education have more freedom to choose a good job with higher salary and more opportunities^10,11^. However, it is difficult to establish the causal nature of these associations because all data came from observational studies that fail to establish causality due to issues such as reverse causation and confounding.

In addition to dispositional factors, increasing attention has been paid to lifestyle factors such as physical activity. We are currently living in a paradoxical time where our society favours strategies to avoid and/or minimise physical effort in several aspects of life; while on the other hand, there is a growing interest and concern for healthy lifestyles emphasising exercises^12^. Literature on the recovery from job stressors has identified physical activity during leisure time among other factors as having a potential to promote recovery by enhancing vigor and mood^13–15^. Furthermore, a systematic review found evidence for a negative relationship between physical activity and a key component of burnout (i.e., exhaustion)^16^. However, recent studies that examined the relationship between physical activity intensity and subjective well-being, found conflicting results^17–19^. Some of the discrepancy between the findings may be due to different study designs (i.e. cross sectional vs prospective) and/or measurement errors. A previous meta-analysis based on a limited number of randomized and controlled intervention studies found mixed results for the positive effect of interventions on job satisfaction^20^. The results from following intervention studies were also mixed^21,22^. This is understandable because all these intervention studies were implemented in small samples and therefore were significantly underpowered. Taken together, the current literature leaves a pending issue regarding the relationship between physical activity and job satisfaction.

In consideration of the above issues, we decided to employ Mendelian randomisation (MR), a technique that helps inferring causality among associations, using genetic information^23^. Causal inference of association between risk factors and outcome can be drawn by assuming that the genetic variants are not associated directly with the outcome but through the risk factor. If the underlying assumptions of MR are satisfied, estimates free from confounding and reverse causality can be obtained^24^. MR makes use of the germ-line genetic variants which are assorted during formation of gametes prior to conception and thus are not confounded by lifestyle or environmental factors in ethnically homogeneous sample of unrelated individuals^25^. Thus, by using MR, it is possible to investigate whether there is a causal association between the exposure and the outcome.

We aimed to understand the casual effect of neuroticism, education, and physical activity on job satisfaction, by conducting MR analyses in UK biobank (UKB) cohort. We tested the hypothesis that observational associations between the studied traits and job satisfaction are causal. To test the hypothesis we employed genetic variants identified in genome wide association studies (GWAS) for their robust association with neuroticism^26^, education^27^ and physical activity^28^. We performed instrumental variable (IV) analyses to investigate the direct effect of each trait on job satisfaction. In addition, since all three traits (neuroticism, education and physical activity) are correlated with each other^11,29–33^, we investigated whether these traits have causal effect on job satisfaction, once the correlation between them has been controlled for.

## Materials and methods

### Study population: The UK Biobank

In this study we used data from UKB that comprised of approximately 500,000 participants aged between 37 and 73 years, recruited during 2006–2010 across the UK. The study has been described in detail elsewhere^34^. Briefly, the resource contains rich phenotypic and health related information on each participant. In addition, whole genome-genotyping data are available for all the participants and was assayed using Applied Biosystems UK BiLEVE Axiom Array (~50,00 participants) and Applied Biosystems UK Biobank Axiom Array platforms (~450,000 participants). The two platforms share 95% of their marker content^34^. Detailed information on the arrays design and content can be found in UK Biobank Axiom Array Content Summary^35^ (35)(35). The genotyping data underwent extensive central quality control (see http://biobank.ctsu.ox.ac.uk) and finally released genotyping data comprising of 805,426 markers from both arrays^34^. Ethical approval for UKB data collection was granted by North-West Multicentre Research Ethics Committee and the use of the data at our Department was further approved by the Regional Ethics Committee of Uppsala, Sweden. All participants provided written consent with the right to withdraw at any time. In the present study, we excluded participants who: withdrew their consent for participation (*n* = 85); had gender mismatch (i.e., genetic sex did not match self-reported sex) (*n* = 373); exhibited genetic relatedness (*n* = 17,284); reported non-British ethnicity, or did not report any ethnicity (*n* = 56,261); had a history of mental/behavioural and neurological disorders according to the ICD-10 classification (*n* = 25,576) and lacking information on neuroticism score (*n* = 72,294). Thus, 315,536 participants were included in the study based on these inclusion/exclusion criteria.

### Phenotypic information

The neuroticism score as reported in UKB was based on twelve neurotic behavioural domains from the Eysenck Personality questionnaire (EPQ-N), and was externally derived by Smith et al.^36^ as the sum of the scores on each domain. The score range from zero to twelve, with higher score corresponding to higher degree of neurotic behaviour.

Information related to education was extracted from the reported highest educational qualification through the questionnaire. We assigned an age at which the person left education based on his/her reported educational level. Assignment of age associated with highest reported qualification for the UKB participants included in this study is given in supplementary table 1. The final education variable ranges from age 15 to 21 years with higher age representing higher educational attainment.

Information regarding physical activity was extracted from the touchscreen-based questions related to the weekly frequency and duration (in minutes) of walking, moderate physical activity and vigorous physical activity. The information was then used to calculate the metabolic equivalent per time (MET) score. First, extreme outliers were excluded (defined as having a z-score out of the range of ±3.29 for of the duration of walking, duration of moderate physical activity and for the duration of vigorous physical activity). MET score (minutes/week) was then derived using the coefficients for each category obtained from the international Physical activity Questionnaire short form, by using the formula: MET score = [(Number of days/week of walking 10 + minutes × Duration of walking × 3.3) + (Number of days/week of moderate physical activity 10+ minutes × Duration of moderate physical activity × 4.0) + (Number of days/week of vigorous physical activity 10+ minutes × Duration of vigorous physical activity × 8)]^37^.

Job satisfaction in UKB was derived from a touchscreen question: "In general how satisfied are you with the WORK that you do?" We recoded the variable as system missing for participants who were unemployed. The final job satisfaction scale ranged from 1 to 6, with higher score representing higher job satisfaction. For all variables, the answers “do not know” or “prefer not to answer” were considered as missing.

Information regarding baseline demographic measures such as age, sex, body mass index (BMI = weight in Kilograms/height in metres^2^) and socioeconomic status were collected at the time of initial assessment in UKB. Townsend deprivation index (TDI; calculated on the basis of participants location in the UK and information from the most recent national census) was used as a proxy for socioeconomic status. Information in UKB has been collected at several centres across the UK and this aspect was taken care of by taking into account the UKB assessment centre at which the participant was consented.

### Genetic variants and genetic risk scores (GRSs)

Genetic variants that had genome wide significant associations (*P* < 5 × 10^−8^) with neuroticism, education and physical activity were identified using the latest published meta-analyses of GWAS in individuals of European ancestry. We identified 136 single nucleotide polymorphisms (SNPs) associated with neuroticism^26^, 74 SNPs from GWAS of educational attainment^27^ and 23 SNPs from GWAS of habitual physical activity^28^. Each SNP was recoded as 0, 1 and 2, depending on the number of trait-specific risk increasing alleles carried by an individual. An unweighted GRS was calculated for each individual in UKB using PLINK software version 1.9^38^. Thus, GRS for neuroticism (GRS_Neuroticism_; comprised of 136 SNPs) ranged from 96 to 166 and corresponded to the number of neuroticism-increasing alleles per individual. Similar to GRS_Neuroticism_, unweighted GRS for education (GRS_Education_; comprised of 70 SNPs (four SNPs were excluded due to non-availability or poor genotype quality (<90%) in UKB)) and unweighted GRS for physical activity (GRS_Physical activity_; comprised of 22 SNPs (one SNP was excluded due to poor genotyping quality in UKB)) were calculated. The GRS_Education_ ranged from 45 to 96 and GRS_Physical activity_ ranged from 7 to 35 risk alleles respectively. We could not calculate the weighted GRS for neuroticism because the weights should be derived from independent samples to avoid introducing bias into the effect estimates^39^ and the GWAS studies for each of the studied traits included the UKB samples^26–28^.

### Statistical analyses

Statistical analyses were performed using STATA (version 13.1, Stata Corp, Texas, USA) and PLINK (version 1.9) software. Prior to analyses, MET score and BMI were log transformed and neuroticism, education and MET score were changed to z-scores for comparability. The associations between the traits and their respective GRSs were tested using linear regression models adjusted for age and sex.

### Observational analyses

We applied two different models in the observational analyses; a univariable (single-variable) model and a multivariable model. In the univariable model, association between the trait-specific z-scores (z-score for neuroticism, education and physical activity) and job satisfaction was assessed using linear regression adjusting for age, sex, UKB assessment centre and TDI. In the multivariable model, z-scores for neuroticism, education and physical activity were included together in the same regression model along with the covariates.

### Mendelian randomization (MR) analyses

**Fig. 1:**
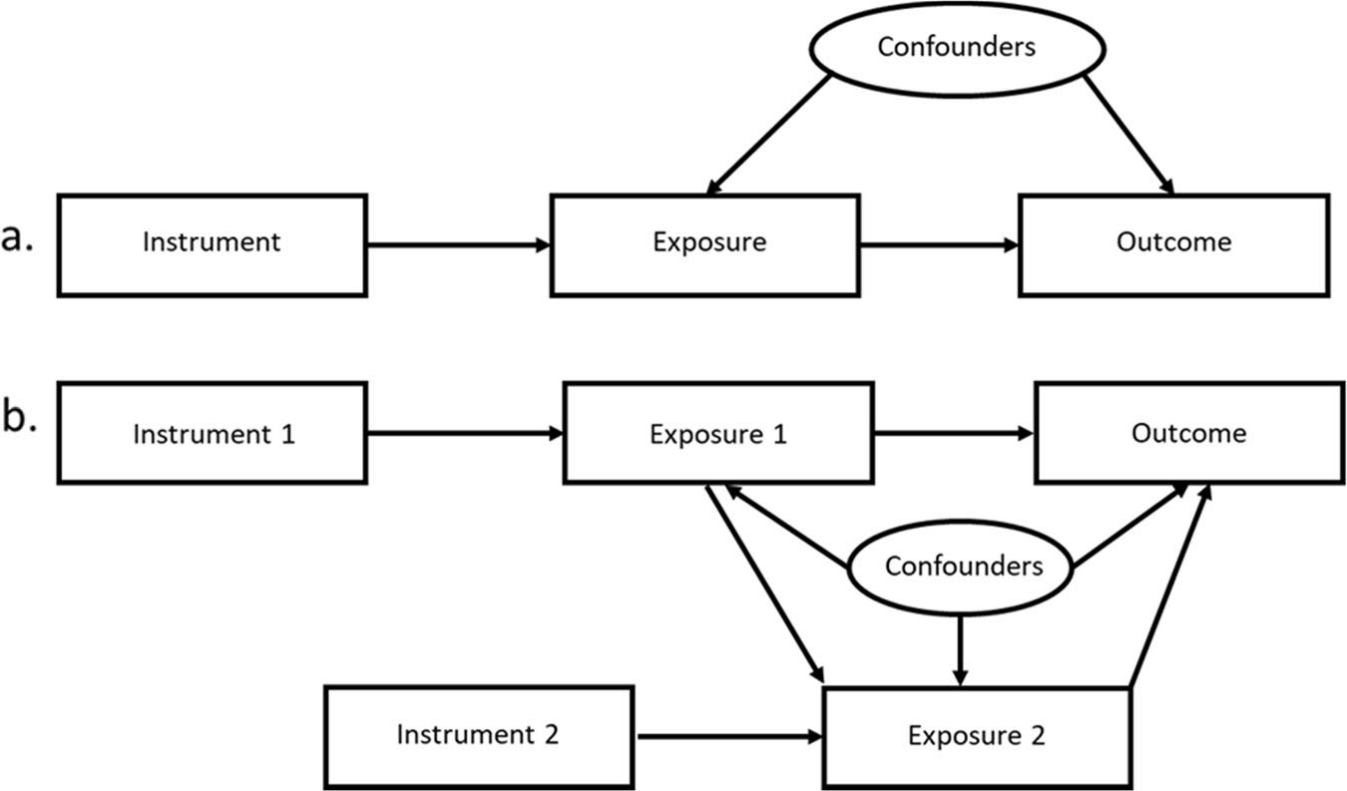
Directed acyclic graph demonstrating (**a**) Standard Mendelian randomization approach and (**b**) Multivariable Mendelian randomization approach using two exposures and two instruments. In the present study, genetic risk score derived from genetic variants associated with their respective exposures (neuroticism, education and physical activity) were used as instruments to assess the causal association between exposures and outcome (job satisfaction).

The MR approach is based on three key assumptions which in the context of this study are (i) each GRS strongly associate with its corresponding trait; (ii) the GRS does not associate with any confounding factor that could bias the observational association between the trait and job satisfaction; and (iii) the GRS associates with job satisfaction only through its effect on the corresponding trait, assuming a linear association between the trait and the job satisfaction (Fig. 1a). We conducted standard (univariable MR) to investigate the total effect of each trait on the job satisfaction and multivariable MR (MVMR) to investigate the direct effect of each trait on the job satisfaction. To conduct univariable MR analyses, we performed Instrumental variable (IV) analyses employing two-stage least square regression (TSLS) method. In the first stage, for each trait, association between the GRS and z-score was assessed using linear regression and predicted fitted values based on the instrument were obtained. In the second stage, linear regression was performed with job satisfaction and genetically predicted exposure level from the first stage. In both stages, analyses were adjusted for age, sex, UKB assessment centre and TDI. For each trait, TSLS was performed using *ivreg2* command in STATA.

### Multivariable Mendelian randomization (MVMR) analyses

To estimate the direct effect of each trait on job satisfaction, we performed MVMR analyses (Fig. 1b). It is an extension of the standard MR analyses (to estimate the effect of multiple exposure variables on an outcome) which can be applied to understand whether all the exposures (neuroticism, education and physical activity) exert a causal effect or whether one mediates the effect of the other on the outcome (job satisfaction)^40^. Similar to standard MR approach, MVMR is also based on the assumptions that genetic variants associate with the exposure variables but do not affect the outcome other than through these variables^41^. To perform MVMR, a multi-stage approach was used. First, for each trait, fitted values were obtained by regressing GRS on the z-score for the corresponding trait. Second, the fitted values of neuroticism, education and physical activity were used together in the same regression model to estimate the direct effect of neuroticism on the job satisfaction^41,42^. All the covariates were included in each stage/regression. MVMR was performed using *ivreg2* command in STATA.

### Sensitivity analyses

In the sensitivity analyses, all the analyses were repeated by additionally adjusting for BMI in the model. We further tested whether each SNP used to create a GRS is a valid instrument in itself. To test this, each trait-specific SNP was regressed on its corresponding trait after adjusting for age and sex and on the job satisfaction after additionally adjusting for the corresponding trait. All the SNPs that did not show nominal association with their corresponding trait (*P* < 0.05) and/or showed nominal significant association with job satisfaction were not taken into consideration for GRS construction. Thus, we excluded 10 SNPs for neuroticism, 11 SNPs for education and 11 SNPs for physical activity and calculated the sensitivity GRS for neuroticism, education and physical activity based on 126, 59 and 11 SNPs, respectively. We repeated all the univariable and MVMR analyses using sensitivity GRS.

### Testing instruments strength

To assess the strength of the instruments, we calculated the standard F-statistic for neuroticism, education and physical activity and the Sanderson-Windmeijer partial F-statistic (S-W F-statistic)^43^ for the MVMR analysis.

### Power calculations

Statistical power calculations for the study were performed using mRnd, a publically available tool (http://cnsgenomics.com/)^44^.

## Results

We undertook MR analyses on individual level data from 315,536 unrelated, white-British participants from UKB with both genotypic and phenotypic information. Mean age of the study participants was 57 years (standard deviation (SD): 8.0 years) and 53% of the participants were women. Study descriptives are provided in supplementary table 2. In this study we have estimated the effect of multiple related exposures, i.e., neuroticism, education and physical activity on job satisfaction and applied MVMR approach in addition to standard MR because MVMR is particularly useful when the exposures are closely related and provide consistent estimates even in the presence of measurement error in the exposure variables^41^.

### Association between GRSs and traits

Each GRS was strongly associated with its respective trait, i.e. neuroticism, education or physical activity (*P* < 0.001) and was normally distributed in the UKB study sample. GRS_Neuroticism_, GRS_Education_ and GRS_Physical activity_ explain 1.14, 0.63 and 0.03% variance in their respective traits (Table 1).

**Table 1:** Association between genetic risk scores (GRS) and their respective traits in the UKB.

GRS	Beta	SE	*P*-value	Variance explained (%)
**GRS**_**Neuroticism**_	0.014	0.0002	<0.001	1.14
**GRS**_**Education**_	0.015	0.0003	<0.001	0.63
**GRS**_**Physical activity**_	0.006	0.0007	<0.001	0.03

Effect per 1 SD of the corresponding trait. Adjusted for age and sex

### Observational analyses

**Fig. 2:**
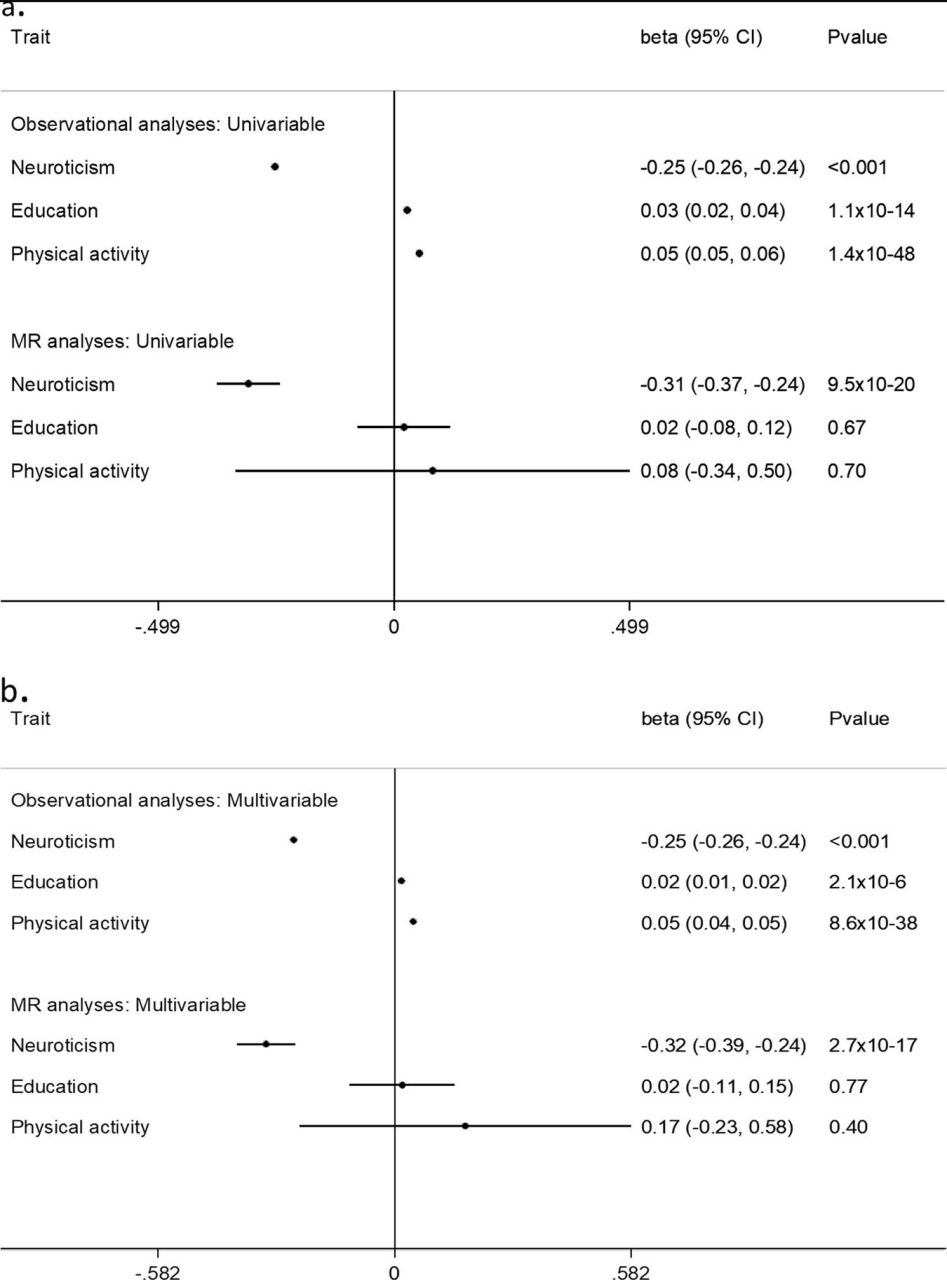
Observational and causal risk estimates using (**a**) Univariable model and (**b**) Multivariable model for 1 standard deviation increase in each trait. All estimates are adjusted for age, sex, UKB assessment centre and Townsend deprivation index. CI: confidence interval.

Data on job satisfaction was available for 73,296 participants after the exclusion of participants who were not employed. In the univariable regression model, there was a strong evidence of observational association between each of the studied trait and job satisfaction. Higher neuroticism score was significantly associated with lower job satisfaction (*β*(95% confidence interval (CI)) = −0.25(−0.26 to −0.24); *P* < 0.001) while higher education status and higher level of physical activity were associated with significantly higher job satisfaction (0.03(0.02 to 0.04); *P* = 1.1 × 10^−14^) and 0.05(0.05 to 0.06); *P* = 1.4 × 10^−48^, respectively) (Fig. 2a). In the multivariable regression model, similar results were obtained. One standard deviation (1 SD) (3.24 units) increase in the neuroticism score was associated with −0.25 (−0.26 to −0.24) units lower job satisfaction (*P* < 0.001). Similarly, a 1 SD higher education level (2.39 years) and 1 SD higher physical activity level (MET score 2699) was associated with higher job satisfaction (*β*(95% CI): 0.02(0.01 to 0.02); 2.1 × 10^−6^ and 0.05 (0.04 to 0.05); 8.6 × 10^−38^, respectively) (Fig. 2b).

### MR analyses

In the MR analyses, a strong evidence for causal association between neuroticism and job satisfaction was observed. In univariable analyses, a 1 SD genetically higher neuroticism score was associated with lower job satisfaction (−0.31(−0.37 to −0.24); 9.5 × 10^−20^). Similarly in the MVMR analyses, a 1 SD genetically higher neuroticism score was independently associated with lower job satisfaction (−0.32(−0.39 to −0.24); 2.7 × 10^−17^). In contrast to the observational analyses, no evidence of causal association between education status and job satisfaction was observed in univariable MR (0.02(−0.08 to 0.12); 0.67) as well as in MVMR (0.02(−0.011 to 0.15); 0.77) analyses. Similarly, physical activity level did not show any causal association with job satisfaction in univariable MR (0.08(−0.34 to 0.50); 0.70) or MVMR (0.17(−0.23 to 0.58); 0.40) analyses (Fig. 2a, b).

### Sensitivity analyses

In the sensitivity analyses, results remained substantially unchanged, when the analyses were additionally adjusted for BMI (supplementary tables 3, 4). Moreover, to test the validity of each SNP used in the GRS for each trait, regression analyses were performed to check associations between SNPs and the exposure, and between SNPs and the outcome (supplementary table 5a–c). In both univariable MR and MVMR analyses with the sensitivity scores, causal association between neuroticism and job satisfaction remain unaffected and additional adjustment of the models with BMI did not affect the results (supplementary table 6a, b). However, in the sensitivity MVMR analyses of physical activity with job satisfaction, physical activity showed positive independent causal association with job satisfaction (0.33(0.03 to 0.64); 0.031) and this association remained unchanged upon additional adjustment with BMI (0.35 (0.03 to 0.68); 0.032) (supplementary table 6a, b).

### Power calculation

Power calculation was performed using the online tool (http://cnsgenomics.com/shiny/mRnd/). Assuming that the genetic instrument for neuroticism explains 1.14% of the variation in the exposure and with the large sample size, we had 100% power to detect an estimate of 0.30 on job satisfaction per SD increase in the neuroticism (supplementary data).

## Discussion

In the present study, by using GRSs as unconfounded proxies for neuroticism, education and physical activity, we attempted to disentangle relationships between these traits and job satisfaction. Using two complementary MR approaches, we found evidence indicating that a high neuroticism score has a causal effect on poor job satisfaction and this relationship is independent of educational level or physical activity. Furthermore, sensitivity analyses highlighted an independent causal association between physical activity and job satisfaction, suggesting a protective causal effect of physical activity on well-being at the working place, even in the presence of a strong negative impact of personality.

Our results confirm that high neuroticism scores lead to low job satisfaction, which has been suggested in previous observational studies^45–47^. A key contribution of this study is the investigation of neuroticism and job satisfaction in a large representative sample of white European population, in contrast to the small observational studies that have investigated this relationship in smaller subgroups such as among bank employees (*N* = 126)^48^, athletic trainers (*N* = 202)^47^, government sector employees (*N* = 399)^45^ and nurses (*N* = 140)^46^. The results from the MVMR analyses highlight that increasing neuroticism decreases job satisfaction. Interestingly, the size of this association decreased only slightly when education and physical activity were taken into account, suggesting that individuals with high neuroticism scores are more likely to become unsatisfied at jobs, independent of their educational level or physical activity.

In line with earlier evidence from observational studies^21^, behaviourally measured physical activity showed strong positive associations with job satisfaction. Although, we did not observe any significant causal association between these two variables in the main MR analyses, we did observe a positive causal association in the sensitivity MR analyses. The lack of association between genetically determined physical activity and job satisfaction can be attributed to the low power of GRS_Physical activity_ as it explains only 0.03% of the total variation in physical activity. Additionally, an inspection of F-statistic revealed that in the sensitivity MR analyses, when the invalid SNPs were removed from the calculated GRS_Physical activity_, the F-statistic was substantially increased from 16 to 39 in univariable MR and from 16 to 32 in MVMR analyses. Therefore, our findings suggests that physically active lifestyle may be beneficial for promoting job satisfaction, as regular physical activity facilitates psychological detachment from work which in turn reduces the risk of prolonged stress responses such as burnout^49,50^. Regular physical activity may also enhance a person’s self-efficacy enabling him/her to feel more competent in coping with everyday work tasks and find them less demanding^16,51^. Lower perceived demands may contribute to lower fatigue and ultimately to higher job satisfaction. Moreover, regular physical activity helps to better handle the psychological stress^52^. The findings of this study show that higher general physical activity is associated with higher job satisfaction. Previous evidence has shown that workplace is a very suitable context to promote and increase employees’ physical activity^53,54^. Taken together, these elements highlight the importance of implementing physical activity programmes at the workplace to ensure positive well-being of employees or providing incentives, such as subsidies for gym, to encourage engagement in regular physical activity. In addition, workplace physical activity interventions have also been connected to many other positive outcomes such as work attendance, interpersonal relationship, and physical health and fitness^20,55^.

Observational studies on the association between education and job satisfaction have yielded conflicting results. For example, a review examining the factors that influence job satisfaction among participants of the nurse residency programme provided mixed results regarding the evidence for improved job satisfaction with increasing nurses education^56^. To the best of our knowledge, our study is the first to evaluate the association between education status and job satisfaction using GRS as an instrument to overcome bias and confounding found in observation studies using a large sample. Our MR analyses suggest no causal association between education and job satisfaction. We suspect that high education may not directly lead to increased job satisfaction because people with higher level of education would also have higher expectation or be over-qualified for certain jobs. There is evidence showing a negative association between over-qualification and job satisfaction^10,57^. Therefore, higher education may result in better job satisfaction but only when there is a good person-job fit^11^.

The strengths of the present study include large sample size comprising of a homogenous population so the results can be translated to the general European population. However, there are certain limitations of our study that should be mentioned. First, the identified causal effects are less biased when weighted GRS is used^58^. We were unable to use a weighted GRS as the weights should be identified in an independent sample to avoid introducing bias into effect estimates^39^ and the UKB constitute a large part of the discovery sample in the GWAS of neuroticism, education attainment and physical activity. Second, we were unable to test the robustness of our findings by applying various methods of MR that employ two-sample approach, as so far no summary statistics are available for job satisfaction. Third, for the same reason stated above, we were unable to investigate the associations in both directions, i.e., we were able to test the association from neuroticism to job satisfaction but not from job satisfaction to neuroticism. Last, an important limitation concerns a power issue, as only a limited proportion of variance of the studied exposure traits can be explained by the respective GRSs, especially for physical activity. Therefore, it is important to emphasise that the negative results in the present study does not necessarily represent lack of causality, but instead stronger non-pleiotropic genetic instruments may be required in order to reliably confirm true absence of association.

In conclusion, we have provided strong evidence that neuroticism is a causal risk factor for job dissatisfaction. This result is consistent with the findings from the observational studies in psychological domain. The main implication of our finding is that reducing the degree of neuroticism will tend to increase job satisfaction resulting in positive effects on the health of the employees and productivity of the companies. Additionally, promoting physically active lifestyle may also positively affect satisfaction at the workplace. Our findings that both neuroticism and physical activity can affect job satisfaction suggest that a combined intervention targeting both lifestyle and psychological aspects may prove to be very effective, e.g., employees could be evaluated for their personality traits (e.g. neuroticism) and allocated to specific physical activity programmes to improve their compliance and obtain beneficial health effects. The findings of this study can prove to be very helpful for researchers, planners and other public health decision makers as our study highlight the additional benefits of measuring dispositional variables such as neuroticism. Especially future studies aiming at examining the relationship between physical activity and work-related subjective well-being (e.g., in future workplace physical activity programmes) may consider the confounding effect of neuroticism.

## Supplementary information

Supplementary data

## Data Availability

The code used for data processing and analysis throughout the current study are available from the corresponding author upon request.
