# Supplementary material for: Personality, lifestyle and job satisfaction: causal association between neuroticism and job satisfaction using Mendelian randomisation in the UK biobank cohort
Source: Transl Psychiatry. 2020 Jan 21;10:11. doi: 10.1038/s41398-020-0691-3 (PMC7026032; doi:10.1038/s41398-020-0691-3)
Supplement: Supplementary file 1 — Supplementary data [file 41398_2020_691_MOESM1_ESM.docx]

**SUPPLEMENTARY DATA**

**Power calculation:**

In order to calculate the power for performing a Mendelian Randomization (MR) study by employing a genetic instrument Z (a SNP or allele score), a continuous exposure variable X and a continuous outcome variable Y , we used the online tool “mRnd: Power calculations for Mendelian Randomization” available online at <http://cnsgenomics.com/shiny/mRnd/>.

We calculated the power of the study using the following seven parameters:

1. Sample size: 73296 (based on availability of information for job satisfaction)
2. Type-I error rate (α): 0.05
3. The regression coefficient for the true underlying causal association between the exposure *(X)* and the outcome *(Y)* variables (β_yx_): 0.31
4. The regression coefficient for the observational association between the exposure *(X)* and outcome *(Y)* variables (β_OLS_): 0.25
5. Proportion of variance explained for the association between the genetic risk score (Z) and the exposure variable (X) (R^2^_xz_): 0.011
6. Variance of the exposure variable (X) (δ^2^(x)): 1
7. Variance of the outcome variable (Y) (δ^2^(y)): 0.083

*Two-stage least squares*

Power calculation for two-stage least squares Mendelian Randomization study using Genetic Risk Score for Neuroticism as genetic instrument (Z), Neuroticism-score as a continuous exposure variable (X) and Job Satisfaction as a continuous outcome variable (Y), is as follows:

| Power | 100% |
| --- | --- |
| Non-Centrality-Parameter (NCP) | 4384.76 |
| The strength of the instrument (F-statistic) | 1113.23 |

*YZ association*

Power calculation for the regression association of Genetic Risk Score for Neuroticism (Z), with Job Satisfaction (Y), is as follows:

| Power | 100% |
| --- | --- |
| Non-Centrality-Parameter (NCP) | 1290.04 |

**Supplementary Table 1.** Educational qualifications

| **Highest educational qualification** | **age completed education** | **Proportion of final sample (N/%)** |
| --- | --- | --- |
| None | 15 | 50153/ 15.9% |
| CSE's/O levels/GCSEs | 16 | 86504/27.4% |
| NVQ/HND/HNC | 18 | 20533/6.5% |
| A levels | 18 | 36430/11.5% |
| Other professional qualifications (e.g. Nursing/Teaching etc) | 20 | 16141/5.1% |
| College/University degree | 21 | 103429/32.8% |

Age associated with highest reported education qualification for the individuals from UKB included in this study

**Supplementary Table 2.** Study specific descriptives of the UK Biobank cohort

| **Trait** | **Data available (N)** | **All** |
| --- | --- | --- |
| Number of participants,  N (%) | 315536 | 315536 (100%) |
| Age in years,  Mean(SD) | 315536 | 56.6 (8.00) |
| Townsend deprivation index, Mean (SD) | 315171 | -1.63 (2.89) |
| BMI (kg/m^2^),  Mean (SD) | 314696 | 27.31 (4.66) |
| Neuroticism score,  Mean (SD) | 315536 | 4.07 (3.24) |
| Job satisfaction score,  Mean (SD) | 73296 | 4.43 (0.87) |
| Age completed education , Mean (SD) | 313190 | 18.06 (2.39) |
| Physical activity (MET score),  Mean (SD) | 249427 | 2618.02 (2699.43) |

**Supplementary Table 3.** Estimates of the Observational association of neuroticism, education and physical activity with job satisfaction after additionally adjusting for BMI

| **Model** | **Participants** | **Beta** | **SE** | **95% Confidence interval** | | ***P*-value** |
| --- | --- | --- | --- | --- | --- | --- |
|  |  |  |  | **Lower CI** | **Upper CI** |  |
| Single variable regression | Neuroticism | -0.252 | 0.004 | -0.259 | -0.245 | <0.001 |
|  | Education | 0.028 | 0.004 | 0.021 | 0.035 | 3.3x10^-14^ |
|  | Physical activity | 0.055 | 0.004 | 0.048 | 0.062 | 7.0x10^-49^ |
| Multivariable regression | Neuroticism | -0.249 | 0.004 | -0.256 | -0.242 | <0.001 |
|  | Education | 0.017 | 0.004 | 0.010 | 0.024 | 1.2x10^-6^ |
|  | Physical activity | 0.047 | 0.004 | 0.040 | 0.054 | 1.2x10^-38^ |

Adjusted for age, sex, UKB assessment centre, Townsend deprivation index and BMI. SE: standard error; CI: confidence interval.

**Supplementary Table 4.** Estimates of the genetic association of neuroticism, education and physical activity with job satisfaction (Mendelian randomization (MR) analyses) after additionally adjusting for BMI

| Model | **Instrument** | **Beta** | **SE** | **95% Confidence interval** | | ***P*-value** | **F-statistic** | **S-W F-statistic** |
| --- | --- | --- | --- | --- | --- | --- | --- | --- |
|  |  |  |  | **Lower CI** | **Upper CI** |  |  |  |
| Single variable  MR | Neuroticism | -0.309 | 0.034 | -0.376 | -0.242 | 9.8x10^-20^ | 640.35 | -- |
|  | Education | 0.017 | 0.053 | -0.087 | 0.120 | 0.75 | 283.32 | -- |
|  | Physical activity | 0.093 | 0.222 | -0.341 | 0.527 | 0.68 | 15.40 | -- |
| Multivariable MR | Neuroticism | -0.312 | 0.038 | -0.386 | -0.238 | 1.4x10^-16^ | 214.37 | 352.74 |
|  | Education | 0.034 | 0.083 | -0.130 | 0.197 | 0.69 | 107.19 | 22.8 |
|  | Physical activity | 0.192 | 0.218 | -0.235 | 0.620 | 0.38 | 13.42 | 14.86 |

Adjusted for age, sex, UKB assessment centre, Townsend deprivation index and BMI. SE: standard error; CI: confidence interval; S-W: Sanderson-Windmeijer.

**Supplementary Table 5a.** Association of 136 GWAS identified neuroticism SNPs with neuroticism and job satisfaction in UKB

| **SNP_ EA** | **Neuroticism** | | | | **Job satisfaction** | | | |
| --- | --- | --- | --- | --- | --- | --- | --- | --- |
|  | **N** | **beta** | **se** | **Pvalue^a^** | **N** | **beta** | **se** | **Pvalue^b^** |
| rs12137398_T | 310144 | 0.04 | 0.01 | 0.00 | 71988 | 0.00 | 0.01 | 0.85 |
| rs11576073_A | 315536 | 0.05 | 0.01 | 0.00 | 73296 | 0.00 | 0.00 | 0.54 |
| rs12747763_T | 315536 | 0.04 | 0.01 | 0.00 | 73296 | 0.01 | 0.01 | 0.40 |
| rs17096778_A | 311850 | 0.11 | 0.02 | 0.00 | 72497 | 0.01 | 0.01 | 0.43 |
| rs11184994_T | 315040 | 0.04 | 0.01 | 0.00 | 73172 | 0.00 | 0.00 | 0.76 |
| rs7536102_T | 315418 | 0.05 | 0.01 | 0.00 | 73265 | 0.00 | 0.00 | 0.62 |
| rs199933_A | 309757 | 0.04 | 0.01 | 0.00 | 71929 | 0.00 | 0.01 | 0.99 |
| rs2488401_C | 307185 | 0.05 | 0.01 | 0.00 | 71408 | 0.00 | 0.01 | 0.39 |
| rs17432675_T | 306249 | 0.05 | 0.01 | 0.00 | 71088 | -0.01 | 0.00 | 0.06 |
| rs17502858_A | 315377 | 0.08 | 0.02 | 0.00 | 73259 | -0.02 | 0.01 | 0.12 |
| rs4396680_A | 315536 | 0.05 | 0.01 | 0.00 | 73296 | 0.00 | 0.01 | 0.70 |
| *rs4665244_A | 315419 | 0.04 | 0.01 | 0.00 | 73267 | -0.01 | 0.00 | 0.05 |
| rs10188070_G | 312282 | 0.05 | 0.01 | 0.00 | 72514 | 0.00 | 0.00 | 0.54 |
| rs11127043_T | 315396 | 0.05 | 0.01 | 0.00 | 73259 | 0.01 | 0.01 | 0.29 |
| rs10048736_G | 314493 | 0.04 | 0.01 | 0.00 | 73031 | 0.00 | 0.00 | 0.58 |
| rs2042555_A | 314592 | 0.06 | 0.01 | 0.00 | 73080 | 0.00 | 0.00 | 0.88 |
| *rs1226574_G | 315371 | 0.05 | 0.01 | 0.00 | 73260 | 0.01 | 0.01 | 0.01 |
| rs1018154_G | 315501 | 0.05 | 0.02 | 0.00 | 73289 | -0.01 | 0.01 | 0.54 |
| rs10497655_T | 315536 | 0.05 | 0.01 | 0.00 | 73296 | 0.00 | 0.00 | 0.92 |
| rs138218528_T | 302014 | 0.05 | 0.01 | 0.00 | 70151 | 0.00 | 0.00 | 0.79 |
| rs78720888_C | 315491 | 0.06 | 0.01 | 0.00 | 73285 | -0.01 | 0.01 | 0.08 |
| rs6737187_G | 315536 | 0.04 | 0.01 | 0.00 | 73296 | 0.00 | 0.00 | 0.58 |
| rs9867227_A | 309337 | 0.06 | 0.01 | 0.00 | 71862 | 0.00 | 0.00 | 0.48 |
| *rs1542212_G | 312043 | 0.06 | 0.01 | 0.00 | 72504 | -0.01 | 0.00 | 0.01 |
| rs6776145_C | 314972 | 0.06 | 0.01 | 0.00 | 73181 | -0.01 | 0.01 | 0.11 |
| rs2015971_C | 310794 | 0.04 | 0.01 | 0.00 | 72162 | 0.00 | 0.00 | 0.86 |
| rs9852417_C | 311058 | 0.04 | 0.01 | 0.00 | 72256 | 0.00 | 0.00 | 0.87 |
| rs9870448_A | 315150 | 0.04 | 0.01 | 0.00 | 73197 | -0.01 | 0.01 | 0.25 |
| rs836927_A | 305652 | 0.06 | 0.01 | 0.00 | 71042 | 0.00 | 0.00 | 0.63 |
| rs59382200_A | 311805 | 0.04 | 0.01 | 0.00 | 72428 | 0.00 | 0.00 | 0.61 |
| rs655836_T | 314974 | 0.05 | 0.01 | 0.00 | 73162 | -0.01 | 0.00 | 0.19 |
| rs4585149_C | 310430 | 0.05 | 0.01 | 0.00 | 72139 | 0.00 | 0.01 | 0.84 |
| rs4697241_G | 312098 | 0.04 | 0.01 | 0.00 | 72466 | 0.01 | 0.00 | 0.07 |
| rs9291211_A | 313919 | 0.04 | 0.01 | 0.00 | 72916 | 0.00 | 0.01 | 0.73 |
| rs6828271_T | 314385 | 0.04 | 0.01 | 0.00 | 73047 | 0.01 | 0.00 | 0.21 |
| rs57360718_C | 310421 | 0.07 | 0.02 | 0.00 | 72140 | 0.01 | 0.01 | 0.15 |
| rs1595693_T | 311685 | 0.05 | 0.01 | 0.00 | 72367 | 0.00 | 0.00 | 0.61 |
| rs17611770_T | 312076 | 0.04 | 0.01 | 0.00 | 72482 | 0.00 | 0.01 | 0.65 |
| rs13168838_G | 315060 | 0.04 | 0.01 | 0.00 | 73186 | 0.00 | 0.00 | 0.61 |
| rs1501673_A | 315137 | 0.07 | 0.01 | 0.00 | 73208 | -0.01 | 0.01 | 0.27 |
| rs11240962_A | 312207 | 0.06 | 0.01 | 0.00 | 72481 | 0.00 | 0.01 | 0.40 |
| rs11740246_T | 312681 | 0.05 | 0.01 | 0.00 | 72608 | -0.01 | 0.01 | 0.36 |
| rs4868748_G | 315147 | 0.04 | 0.01 | 0.00 | 73197 | 0.00 | 0.00 | 0.45 |
| rs2031595_C | 314268 | 0.04 | 0.01 | 0.00 | 73001 | 0.00 | 0.00 | 0.91 |
| rs2071303_T | 315536 | 0.05 | 0.01 | 0.00 | 73296 | 0.00 | 0.00 | 0.79 |
| rs200965_G | 313171 | 0.06 | 0.01 | 0.00 | 72723 | 0.01 | 0.01 | 0.31 |
| *rs28986304_C | 315511 | 0.06 | 0.01 | 0.00 | 73288 | -0.02 | 0.01 | 0.01 |
| rs2503775_A | 315113 | 0.07 | 0.01 | 0.00 | 73206 | 0.00 | 0.01 | 0.52 |
| rs240769_C | 309448 | 0.04 | 0.01 | 0.00 | 71817 | 0.00 | 0.00 | 0.94 |
| rs2056477_G | 314486 | 0.05 | 0.01 | 0.00 | 73054 | 0.00 | 0.01 | 0.83 |
| *rs11509880_A | 314488 | 0.04 | 0.01 | 0.00 | 73062 | -0.01 | 0.00 | 0.03 |
| rs6948810_T | 312758 | 0.04 | 0.01 | 0.00 | 72631 | 0.00 | 0.00 | 0.92 |
| rs10274968_T | 314938 | 0.04 | 0.01 | 0.00 | 73155 | 0.00 | 0.00 | 0.53 |
| rs274632_C | 314461 | 0.05 | 0.01 | 0.00 | 73031 | 0.00 | 0.00 | 0.30 |
| rs35763039_T | 314480 | 0.04 | 0.01 | 0.00 | 73036 | 0.00 | 0.00 | 0.89 |
| rs35048193_T | 315521 | 0.05 | 0.01 | 0.00 | 73294 | -0.01 | 0.00 | 0.14 |
| rs11766965_C | 308713 | 0.04 | 0.01 | 0.00 | 71630 | 0.00 | 0.00 | 0.30 |
| rs3812479_A | 314322 | 0.02 | 0.01 | 0.00 | 72997 | 0.00 | 0.00 | 0.85 |
| rs2407746_G | 311138 | 0.06 | 0.01 | 0.00 | 72242 | 0.01 | 0.00 | 0.16 |
| *rs2921036_T | 309741 | 0.08 | 0.01 | 0.00 | 71894 | 0.01 | 0.00 | 0.01 |
| rs7818437_C | 306598 | 0.08 | 0.01 | 0.00 | 71127 | 0.01 | 0.01 | 0.26 |
| rs28639817_G | 310480 | 0.06 | 0.01 | 0.00 | 72089 | 0.01 | 0.01 | 0.29 |
| rs76288073_C | 315138 | 0.06 | 0.01 | 0.00 | 73204 | 0.00 | 0.01 | 0.43 |
| *rs2737219_A | 314442 | 0.04 | 0.01 | 0.00 | 73045 | -0.01 | 0.00 | 0.01 |
| rs13262595_A | 315092 | 0.04 | 0.01 | 0.00 | 73181 | 0.00 | 0.00 | 0.37 |
| rs2380937_T | 313541 | 0.04 | 0.01 | 0.00 | 72806 | 0.00 | 0.00 | 0.70 |
| rs62550480_C | 315536 | 0.06 | 0.01 | 0.00 | 73296 | 0.01 | 0.01 | 0.23 |
| rs10811883_C | 303850 | 0.04 | 0.01 | 0.00 | 70598 | -0.01 | 0.00 | 0.19 |
| rs10119773_G | 300617 | 0.05 | 0.01 | 0.00 | 69860 | 0.00 | 0.00 | 0.84 |
| rs12344656_G | 312048 | 0.05 | 0.01 | 0.00 | 72513 | 0.00 | 0.00 | 0.62 |
| rs78046549_T | 315486 | 0.06 | 0.01 | 0.00 | 73285 | 0.00 | 0.01 | 0.47 |
| rs75614054_T | 313785 | 0.09 | 0.01 | 0.00 | 72882 | -0.01 | 0.01 | 0.23 |
| rs7025144_T | 312930 | 0.05 | 0.01 | 0.00 | 72665 | 0.01 | 0.00 | 0.30 |
| rs60393230_G | 311974 | 0.05 | 0.01 | 0.00 | 72466 | 0.00 | 0.00 | 0.67 |
| rs11256385_C | 314832 | 0.04 | 0.01 | 0.00 | 73132 | 0.00 | 0.00 | 0.65 |
| rs2093623_G | 305588 | 0.04 | 0.01 | 0.00 | 70899 | 0.00 | 0.00 | 0.79 |
| *rs3740393_G | 314131 | 0.06 | 0.01 | 0.00 | 72937 | 0.01 | 0.01 | 0.02 |
| rs860626_T | 307302 | 0.04 | 0.01 | 0.00 | 71381 | 0.00 | 0.00 | 0.75 |
| rs4757136_T | 313083 | 0.05 | 0.01 | 0.00 | 72719 | -0.01 | 0.00 | 0.06 |
| rs297343_T | 314152 | 0.05 | 0.01 | 0.00 | 72937 | 0.00 | 0.00 | 0.46 |
| rs10835565_T | 314957 | 0.04 | 0.01 | 0.00 | 73159 | 0.00 | 0.00 | 0.86 |
| rs1806153_T | 314495 | 0.07 | 0.01 | 0.00 | 73050 | 0.00 | 0.01 | 0.85 |
| rs12283653_G | 310335 | 0.04 | 0.01 | 0.00 | 72061 | 0.00 | 0.00 | 0.61 |
| rs7107356_G | 314919 | 0.06 | 0.01 | 0.00 | 73138 | 0.00 | 0.00 | 0.81 |
| rs10896636_G | 309582 | 0.07 | 0.01 | 0.00 | 71855 | 0.00 | 0.00 | 0.91 |
| rs10830220_G | 306492 | 0.04 | 0.01 | 0.00 | 71132 | -0.01 | 0.00 | 0.21 |
| rs10790767_T | 311874 | 0.04 | 0.01 | 0.00 | 72451 | 0.00 | 0.00 | 0.45 |
| rs7111031_A | 314929 | 0.07 | 0.01 | 0.00 | 73163 | -0.01 | 0.00 | 0.17 |
| rs11605020_A | 303934 | 0.04 | 0.01 | 0.00 | 70576 | 0.00 | 0.00 | 0.35 |
| rs167915_A | 311199 | 0.05 | 0.01 | 0.00 | 72209 | 0.00 | 0.00 | 0.93 |
| rs4337088_A | 313775 | 0.04 | 0.01 | 0.00 | 72880 | 0.00 | 0.00 | 0.94 |
| rs9971907_G | 308810 | 0.04 | 0.01 | 0.00 | 71671 | 0.00 | 0.00 | 0.44 |
| rs938496_G | 313780 | 0.04 | 0.01 | 0.00 | 72925 | 0.00 | 0.00 | 0.79 |
| rs11608355_C | 313944 | 0.05 | 0.01 | 0.00 | 72916 | 0.00 | 0.00 | 0.85 |
| rs3741475_A | 314895 | 0.06 | 0.01 | 0.00 | 73155 | -0.01 | 0.01 | 0.07 |
| rs6490177_A | 314101 | 0.06 | 0.01 | 0.00 | 72938 | 0.01 | 0.01 | 0.08 |
| rs12426113_G | 313439 | 0.05 | 0.01 | 0.00 | 72763 | 0.00 | 0.01 | 0.53 |
| rs3124426_C | 315462 | 0.04 | 0.01 | 0.00 | 73283 | 0.01 | 0.01 | 0.21 |
| rs4444227_C | 314329 | 0.05 | 0.01 | 0.00 | 73022 | 0.00 | 0.01 | 0.47 |
| rs1892350_G | 313997 | 0.04 | 0.01 | 0.00 | 72923 | 0.00 | 0.00 | 0.35 |
| rs7999314_T | 311559 | 0.04 | 0.01 | 0.00 | 72363 | 0.00 | 0.00 | 0.31 |
| rs9516861_A | 313204 | 0.06 | 0.01 | 0.00 | 72730 | 0.00 | 0.01 | 0.83 |
| rs3783007_G | 314648 | 0.05 | 0.01 | 0.00 | 73112 | 0.00 | 0.00 | 0.99 |
| rs9521987_A | 313961 | 0.04 | 0.01 | 0.00 | 72928 | -0.01 | 0.00 | 0.24 |
| *rs1778377_T | 313890 | 0.04 | 0.01 | 0.00 | 72926 | -0.01 | 0.00 | 0.02 |
| rs11627348_A | 310153 | 0.05 | 0.01 | 0.00 | 72038 | -0.01 | 0.01 | 0.27 |
| rs1275411_T | 312967 | 0.05 | 0.01 | 0.00 | 72693 | 0.00 | 0.00 | 0.59 |
| rs9972151_C | 314847 | 0.04 | 0.01 | 0.00 | 73128 | 0.00 | 0.00 | 0.98 |
| rs9671386_A | 315536 | 0.06 | 0.01 | 0.00 | 73296 | 0.00 | 0.00 | 0.62 |
| rs12442330_T | 313261 | 0.03 | 0.01 | 0.00 | 72756 | -0.01 | 0.00 | 0.11 |
| rs12898855_G | 306002 | 0.04 | 0.01 | 0.00 | 71044 | 0.00 | 0.00 | 0.93 |
| rs8033552_A | 306672 | 0.04 | 0.01 | 0.00 | 71268 | 0.00 | 0.01 | 0.56 |
| rs4360891_C | 314022 | 0.04 | 0.01 | 0.00 | 72901 | 0.00 | 0.00 | 0.51 |
| rs11852334_T | 311280 | 0.03 | 0.01 | 0.00 | 72272 | 0.00 | 0.00 | 0.37 |
| rs3785232_T | 305479 | 0.05 | 0.01 | 0.00 | 70948 | 0.00 | 0.00 | 0.97 |
| rs12926477_G | 313619 | 0.04 | 0.01 | 0.00 | 72879 | 0.00 | 0.00 | 0.97 |
| rs3751855_T | 315522 | 0.05 | 0.01 | 0.00 | 73295 | 0.00 | 0.00 | 0.76 |
| rs9933638_A | 314397 | 0.04 | 0.01 | 0.00 | 73024 | 0.00 | 0.00 | 0.73 |
| rs1424144_C | 313437 | 0.04 | 0.01 | 0.00 | 72818 | -0.01 | 0.00 | 0.18 |
| rs2042395_G | 312040 | 0.05 | 0.01 | 0.00 | 72445 | 0.00 | 0.01 | 0.95 |
| rs529900_C | 310530 | 0.04 | 0.01 | 0.00 | 72080 | 0.00 | 0.00 | 0.98 |
| *rs12938775_G | 315536 | 0.05 | 0.01 | 0.00 | 73296 | -0.01 | 0.00 | 0.01 |
| rs35982947_A | 307782 | 0.05 | 0.01 | 0.00 | 71430 | 0.00 | 0.00 | 0.67 |
| rs77804065_T | 309796 | 0.09 | 0.01 | 0.00 | 71941 | 0.00 | 0.01 | 0.95 |
| rs1980119_G | 314923 | 0.04 | 0.01 | 0.00 | 73137 | 0.01 | 0.00 | 0.24 |
| rs56084168_C | 314646 | 0.06 | 0.01 | 0.00 | 73110 | 0.01 | 0.01 | 0.15 |
| rs10460051_T | 314984 | 0.05 | 0.01 | 0.00 | 73159 | 0.00 | 0.00 | 0.59 |
| rs11082011_C | 312164 | 0.07 | 0.01 | 0.00 | 72520 | 0.00 | 0.00 | 0.69 |
| rs11875397_T | 313037 | 0.04 | 0.01 | 0.00 | 72709 | 0.00 | 0.01 | 0.54 |
| rs4632195_T | 314120 | 0.05 | 0.01 | 0.00 | 72976 | -0.01 | 0.00 | 0.19 |
| rs12958048_A | 314534 | 0.05 | 0.01 | 0.00 | 73048 | 0.00 | 0.00 | 0.76 |
| rs7240682_C | 312596 | 0.05 | 0.01 | 0.00 | 72615 | -0.01 | 0.01 | 0.31 |
| rs1861777_G | 314617 | 0.04 | 0.01 | 0.00 | 73065 | 0.00 | 0.00 | 0.40 |
| rs62212171_C | 314651 | 0.06 | 0.01 | 0.00 | 73083 | 0.00 | 0.01 | 1.00 |
| rs2425752_T | 315536 | 0.05 | 0.01 | 0.00 | 73296 | 0.00 | 0.00 | 0.85 |
| rs9611519_T | 315443 | 0.05 | 0.01 | 0.00 | 73276 | -0.01 | 0.00 | 0.20 |

^a^adjusted for age and sex. ^b^adjusted for age, sex and neuroticism.*removed from the sensitivity genetic risk score for neuroticism. SNP: single nucleotide polymorphism; EA: Effect Allele.

**Supplementary Table 5b.** Association of 70 GWAS identified education attainment SNPs with age completed education and job satisfaction in UKB

| **SNP_EA** | **Age completed education** | | | | **Job satisfaction** | | | |
| --- | --- | --- | --- | --- | --- | --- | --- | --- |
|  | **N** | **beta** | **se** | **Pvalue^a^** | **N** | **beta** | **se** | **Pvalue^b^** |
| rs301800_T | 313190 | 0.05 | 0.01 | 0.00 | 72929 | 0.00 | 0.01 | 0.76 |
| rs11210860_A | 309943 | 0.04 | 0.01 | 0.00 | 72140 | 0.00 | 0.00 | 0.64 |
| rs34305371_A | 313190 | 0.08 | 0.01 | 0.00 | 72929 | -0.01 | 0.01 | 0.11 |
| rs1008078_C | 309770 | 0.06 | 0.01 | 0.00 | 72111 | -0.01 | 0.00 | 0.06 |
| rs11588857_A | 312945 | 0.06 | 0.01 | 0.00 | 72874 | 0.00 | 0.01 | 0.84 |
| rs1777827_A | 312736 | 0.02 | 0.01 | 0.00 | 72824 | 0.00 | 0.00 | 0.66 |
| rs2992632_A | 301812 | 0.04 | 0.01 | 0.00 | 70287 | 0.00 | 0.01 | 0.73 |
| rs76076331_T | 310345 | 0.06 | 0.01 | 0.00 | 72269 | 0.00 | 0.01 | 0.97 |
| rs11689269_C | 313029 | 0.01 | 0.01 | 0.03 | 72891 | 0.00 | 0.00 | 0.39 |
| rs1606974_A | 312025 | 0.05 | 0.01 | 0.00 | 72664 | 0.00 | 0.01 | 0.98 |
| rs11690172_A | 310851 | 0.02 | 0.01 | 0.00 | 72374 | -0.01 | 0.00 | 0.16 |
| rs2457660_C | 306036 | 0.04 | 0.01 | 0.00 | 71289 | 0.00 | 0.00 | 0.83 |
| rs114598875_G | 307964 | 0.04 | 0.01 | 0.00 | 71758 | 0.00 | 0.01 | 0.61 |
| rs10496091_G | 313190 | 0.03 | 0.01 | 0.00 | 72929 | 0.00 | 0.01 | 0.59 |
| rs13402908_C | 306225 | 0.03 | 0.01 | 0.00 | 71252 | 0.00 | 0.00 | 0.66 |
| rs4851251_C | 305763 | 0.04 | 0.01 | 0.00 | 71101 | 0.00 | 0.01 | 0.90 |
| rs12987662_A | 311065 | 0.05 | 0.01 | 0.00 | 72434 | 0.01 | 0.00 | 0.28 |
| rs17824247_C | 311517 | 0.04 | 0.01 | 0.00 | 72522 | 0.00 | 0.00 | 0.93 |
| rs16845580_T | 308283 | 0.05 | 0.01 | 0.00 | 71768 | 0.00 | 0.00 | 0.73 |
| rs4500960_C | 311119 | 0.03 | 0.01 | 0.00 | 72423 | 0.00 | 0.00 | 0.33 |
| rs6739979_C | 311466 | 0.03 | 0.01 | 0.00 | 72525 | 0.00 | 0.00 | 0.81 |
| rs2245901_G | 310388 | 0.03 | 0.01 | 0.00 | 72267 | 0.00 | 0.00 | 0.49 |
| rs55830725_T | 311619 | 0.03 | 0.01 | 0.00 | 72575 | 0.00 | 0.01 | 0.75 |
| rs35761247_A | 313190 | 0.09 | 0.01 | 0.00 | 72929 | 0.01 | 0.01 | 0.21 |
| rs62259535_A | 313125 | 0.06 | 0.02 | 0.00 | 72915 | 0.01 | 0.01 | 0.53 |
| *rs148734725_A | 312923 | 0.09 | 0.01 | 0.00 | 72879 | 0.01 | 0.00 | 0.04 |
| rs11712056_T | 312228 | 0.08 | 0.01 | 0.00 | 72708 | 0.00 | 0.00 | 0.29 |
| rs112634398_A | 305864 | 0.11 | 0.02 | 0.00 | 71196 | -0.01 | 0.01 | 0.52 |
| rs62263923_G | 304686 | 0.05 | 0.01 | 0.00 | 70952 | 0.00 | 0.00 | 0.77 |
| rs6799130_G | 313082 | 0.02 | 0.01 | 0.00 | 72910 | 0.01 | 0.00 | 0.06 |
| rs12646808_T | 299142 | 0.03 | 0.01 | 0.00 | 69559 | -0.01 | 0.00 | 0.09 |
| *rs34072092_T | 311561 | 0.01 | 0.01 | 0.17 | 72531 | 0.00 | 0.01 | 0.59 |
| *rs3101246_G | 313190 | 0.00 | 0.01 | 0.56 | 72929 | 0.00 | 0.00 | 0.62 |
| rs4863692_T | 312689 | 0.03 | 0.01 | 0.00 | 72789 | -0.01 | 0.00 | 0.26 |
| rs4493682_C | 312698 | 0.02 | 0.01 | 0.00 | 72805 | -0.01 | 0.01 | 0.18 |
| rs2964197_T | 310320 | 0.02 | 0.01 | 0.00 | 72224 | 0.00 | 0.00 | 0.75 |
| rs61160187_G | 309738 | 0.05 | 0.01 | 0.00 | 72110 | 0.00 | 0.00 | 0.64 |
| rs324886_C | 309737 | 0.04 | 0.01 | 0.00 | 72082 | 0.00 | 0.00 | 0.38 |
| rs10061788_A_n | 296908 | 0.06 | 0.01 | 0.00 | 69200 | 0.01 | 0.01 | 0.36 |
| rs2431108_T | 311845 | 0.03 | 0.01 | 0.00 | 72625 | 0.00 | 0.00 | 0.80 |
| *rs1402025_T | 311524 | 0.05 | 0.01 | 0.00 | 72550 | -0.01 | 0.01 | 0.02 |
| rs62379838_T | 312973 | 0.04 | 0.01 | 0.00 | 72876 | 0.00 | 0.00 | 0.46 |
| rs56231335_C | 312344 | 0.03 | 0.01 | 0.00 | 72733 | 0.01 | 0.00 | 0.29 |
| rs9320913_A | 313000 | 0.08 | 0.01 | 0.00 | 72889 | 0.00 | 0.00 | 0.45 |
| *rs7767938_T | 313033 | 0.01 | 0.01 | 0.17 | 72903 | 0.00 | 0.01 | 0.43 |
| *rs2615691_G | 311142 | 0.00 | 0.02 | 0.99 | 72455 | -0.01 | 0.01 | 0.34 |
| rs12531458_A | 307941 | 0.02 | 0.01 | 0.00 | 71675 | 0.00 | 0.00 | 0.28 |
| rs12671937_A | 308742 | 0.03 | 0.01 | 0.00 | 71869 | 0.00 | 0.00 | 0.63 |
| rs113520408_A | 309500 | 0.03 | 0.01 | 0.00 | 72040 | 0.00 | 0.01 | 0.75 |
| rs17167170_A | 310543 | 0.06 | 0.01 | 0.00 | 72287 | 0.00 | 0.01 | 0.64 |
| *rs11768238_G | 309091 | 0.03 | 0.01 | 0.00 | 71914 | -0.01 | 0.00 | 0.03 |
| rs12682297_T | 311755 | 0.03 | 0.01 | 0.00 | 72592 | 0.00 | 0.00 | 0.82 |
| rs1871109_G | 311938 | 0.03 | 0.01 | 0.00 | 72641 | -0.01 | 0.00 | 0.25 |
| rs13294439_C | 312337 | 0.08 | 0.01 | 0.00 | 72720 | 0.00 | 0.00 | 0.36 |
| rs895606_A | 312586 | 0.04 | 0.01 | 0.00 | 72781 | 0.01 | 0.00 | 0.22 |
| rs7854982_C | 307760 | 0.03 | 0.01 | 0.00 | 71605 | 0.00 | 0.00 | 0.54 |
| rs11191193_A | 309198 | 0.05 | 0.01 | 0.00 | 72039 | 0.00 | 0.00 | 0.74 |
| rs12772375_G | 303760 | 0.03 | 0.01 | 0.00 | 70695 | 0.00 | 0.00 | 0.98 |
| rs7945718_A | 308624 | 0.03 | 0.01 | 0.00 | 71852 | -0.01 | 0.00 | 0.06 |
| *rs7955289_A | 311751 | 0.04 | 0.01 | 0.00 | 72596 | -0.01 | 0.00 | 0.04 |
| rs2456973_C | 313190 | 0.04 | 0.01 | 0.00 | 72929 | 0.00 | 0.00 | 0.80 |
| rs7131944_A | 307663 | 0.04 | 0.01 | 0.00 | 71617 | 0.00 | 0.00 | 0.49 |
| *rs572016_A | 312860 | 0.01 | 0.01 | 0.16 | 72851 | 0.00 | 0.00 | 0.47 |
| rs7306755_A | 312568 | 0.05 | 0.01 | 0.00 | 72780 | 0.00 | 0.01 | 0.77 |
| rs9537821_A | 313046 | 0.06 | 0.01 | 0.00 | 72885 | 0.00 | 0.01 | 0.37 |
| rs1043209_A | 313136 | 0.03 | 0.01 | 0.00 | 72913 | 0.01 | 0.00 | 0.22 |
| *rs17119973_G | 306861 | 0.03 | 0.01 | 0.00 | 71454 | 0.01 | 0.01 | 0.03 |
| rs12969294_G | 310396 | 0.06 | 0.01 | 0.00 | 72247 | 0.00 | 0.00 | 0.63 |
| rs2837992_T | 309793 | 0.04 | 0.01 | 0.00 | 72106 | 0.00 | 0.00 | 0.74 |
| *rs165633_G_n | 297347 | 0.01 | 0.01 | 0.43 | 69137 | 0.01 | 0.01 | 0.13 |

^a^adjusted for age and sex. ^b^adjusted for age, sex and age completed education.*removed from the sensitivity genetic risk score for education. SNP: single nucleotide polymorphism; EA: Effect Allele.

**Supplementary Table 5c.** Association of 22 GWAS identified physical activity SNPs with MET score and job satisfaction in UKB

| **SNP_ EA** | **MET score** | | | | **Job satisfaction** | | | |
| --- | --- | --- | --- | --- | --- | --- | --- | --- |
|  | **N** | **beta** | **se** | **Pvalue^a^** | **N** | **beta** | **se** | **Pvalue^b^** |
| *rs1248860_G | 254093 | -0.01 | 0.00 | 0.00 | 60820 | -0.01 | 0.00 | 0.02 |
| *rs2035562_A | 251181 | -0.02 | 0.00 | 0.00 | 60091 | -0.01 | 0.01 | 0.03 |
| *rs62253088_T | 252676 | 0.01 | 0.00 | 0.05 | 60490 | 0.01 | 0.01 | 0.13 |
| *rs159544_G | 251412 | 0.00 | 0.00 | 0.41 | 60196 | 0.01 | 0.01 | 0.23 |
| *rs10946808_G | 254093 | 0.00 | 0.00 | 0.72 | 60820 | -0.01 | 0.01 | 0.13 |
| rs149943_G | 254093 | 0.01 | 0.00 | 0.01 | 60820 | 0.00 | 0.01 | 0.56 |
| *rs3094622_A | 254093 | 0.01 | 0.00 | 0.01 | 60820 | -0.02 | 0.01 | 0.01 |
| rs2764261_A | 251556 | 0.01 | 0.00 | 0.00 | 60215 | 0.00 | 0.01 | 0.81 |
| rs328902_T | 253318 | 0.02 | 0.00 | 0.00 | 60628 | 0.01 | 0.01 | 0.09 |
| rs7791992_A | 252081 | 0.02 | 0.00 | 0.00 | 60288 | 0.01 | 0.01 | 0.20 |
| rs1043595_G | 250978 | 0.01 | 0.00 | 0.00 | 60057 | 0.00 | 0.01 | 0.46 |
| rs7804463_T | 251686 | 0.02 | 0.00 | 0.00 | 60244 | -0.01 | 0.00 | 0.26 |
| rs13243553_G | 251668 | 0.01 | 0.00 | 0.00 | 60225 | 0.00 | 0.01 | 0.74 |
| rs2988004_G_n | 242369 | 0.02 | 0.00 | 0.00 | 58050 | 0.00 | 0.01 | 0.67 |
| *rs3781411_C | 254011 | 0.01 | 0.00 | 0.05 | 60789 | 0.00 | 0.01 | 0.78 |
| *rs75930676_C | 249368 | 0.00 | 0.01 | 1.00 | 59675 | 0.00 | 0.01 | 0.79 |
| rs743580_A | 254093 | 0.01 | 0.00 | 0.01 | 60820 | 0.00 | 0.00 | 0.67 |
| *rs166840_G | 248474 | 0.00 | 0.00 | 0.13 | 59436 | 0.01 | 0.01 | 0.01 |
| *rs55657917_G | 251704 | 0.00 | 0.00 | 0.50 | 60244 | -0.01 | 0.01 | 0.04 |
| rs59499656_T | 251736 | 0.01 | 0.00 | 0.03 | 60236 | 0.01 | 0.01 | 0.19 |
| *rs111901094_G | 246946 | 0.00 | 0.00 | 0.95 | 59121 | -0.01 | 0.01 | 0.20 |
| rs429358_C | 254093 | 0.02 | 0.00 | 0.00 | 60820 | 0.01 | 0.01 | 0.28 |

^a^adjusted for age and sex. ^b^adjusted for age, sex and MET score.*removed from the sensitivity genetic risk score for physical activity. SNP: single nucleotide polymorphism; EA: Effect Allele; MET: metabolic equivalent per time.

**Supplementary Table 6a. Estimates of the genetic association of neuroticism, education and physical activity with job satisfaction (Mendelian randomization (MR) analyses) after removing invalid SNPs from instruments.**

| **Model** | **Instrument** | **Beta** | **SE** | **95% Confidence interval** | | ***P*-value** | **F-statistic** | **S-W F-statistic** |
| --- | --- | --- | --- | --- | --- | --- | --- | --- |
|  |  |  |  | **Lower CI** | **Upper CI** |  |  |  |
| Single variable  MR | Neuroticism | -0.279 | 0.035 | -0.348 | -0.209 | 3.4x10^-15^ | 588.52 | -- |
|  | Education | 0.020 | 0.052 | -0.083 | 0.122 | 0.71 | 288.42 | -- |
|  | Physical activity | 0.253 | 0.143 | -0.027 | 0.533 | 0.076 | 38.49 | -- |
| Multivariable MR | Neuroticism | -0.290 | 0.041 | -0.371 | -0.210 | 1.2x10^-12^ | 197.75 | 376.37 |
|  | Education | 0.045 | 0.066 | -0.084 | 0.173 | 0.50 | 107.36 | 76.00 |
|  | Physical activity | 0.334 | 0.154 | 0.031 | 0.636 | 0.031 | 16.70 | 32.07 |

Adjusted for age, sex, UKB assessment centre, Townsend deprivation index. SE: standard error; CI: confidence interval; S-W F-statistic : Sanderson-Windmeijer F-statistic.

**Supplementary Table 6b.** Estimates of the genetic association of neuroticism, education and physical activity with job satisfaction (Mendelian randomization (MR) analyses) after removing invalid SNPs from instruments and additionally adjusting for BMI.

| **Model** | **Instrument** | **Beta** | **SE** | **95% Confidence interval** | | ***P*-value** | **F-statistic** | **S-W F-statistic** |
| --- | --- | --- | --- | --- | --- | --- | --- | --- |
|  |  |  |  | **Lower CI** | **Upper CI** |  |  |  |
| Single variable  MR | Neuroticism | -0.279 | 0.035 | -0.349 | -0.210 | 3.4x10^-15^ | 587.14 | -- |
|  | Education | 0.016 | 0.055 | -0.093 | 0.124 | 0.78 | 258.90 | -- |
|  | Physical activity | 0.259 | 0.146 | -0.027 | 0.546 | 0.076 | 37.46 | -- |
| Multivariable MR | Neuroticism | -0.284 | 0.042 | -0.365 | -0.202 | 8.9x10^-12^ | 197.15 | 350.97 |
|  | Education | 0.072 | 0.079 | -0.083 | 0.227 | 0.36 | 98.56 | 48.92 |
|  | Physical activity | 0.354 | 0.165 | 0.030 | 0.677 | 0.032 | 18.66 | 28.26 |

Adjusted for age, sex, UKB assessment centre, Townsend deprivation index and BMI. SE: standard error; CI: confidence interval; S-W: Sanderson-Windmeijer.
